# Supplementary material for: Discovery of GOT1 Inhibitors from a Marine-Derived Aspergillus terreus That Act against Pancreatic Ductal Adenocarcinoma
Source: Mar Drugs. 2021 Oct 20;19(11):588. doi: 10.3390/md19110588 (PMC8618880; doi:10.3390/md19110588)

Discovery of GOT1 Inhibitors from a Marine-derived *Aspergillus terreus* that Against Pancreatic Ductal Adenocarcinoma

Shan Yan,[1,†] Changxing Qi,[1,†] Wei Song,[2] Qianqian Xu,[1] Weiguang Sun,[1,*] Yonghui Zhang[1,*]

[1]Hubei Key Laboratory of Natural Medicinal Chemistry and Resource Evaluation, School of Pharmacy, Tongji Medical College, Huazhong University of Science and Technology, Wuhan 430030, People's Re-public of China; yanshanwhu@163.com (S.Y.); qichangxing@hust.edu.cn (C.Q.); Xuxuqq@hust.edu.cn (Q.X.).

[2]Guangdong Provincial Key Laboratory of Microbial Culture Collection and Application, State Key La-boratory of Applied Microbiology Southern China, Institute of Microbiology, Guangdong Academy of Sciences, Guangzhou 510000, China; 20191103@zcmu.edu.cn (W.S.).

[*]Correspondence: zhangyh@mails.tjmu.edu.cn (Y.Z.); zhuhucheng@hust.edu.cn (H.Z.).

[†]S.Y. and C.Q. contributed equally to this work.

ITS sequence of the fungus

CTTCCGGTAGGGTGAACCTGCGGAAGGATCATTACCGAGTGCGGGTCTT
TATGGCCCAACCTCCCACCCGTGACTATTGTACCTTGTTGCTTCGGCGGGCC
CGCCAGCGTTGCTGGCCGCCGGGGGGCGACTCGCCCCCGGGCCCGTGCCC
GCCGGAGACCCCAACATGAACCCTGTTCTGAAAGCTTGCAGTCTGAGTGT
GATTCTTTGCAATCAGTTAAAACTTTCAACAATGGATCTCTTGGTTCCGGCA
TCGATGAAGAACGCAGCGAAATGCGATAACTAATGTGAATTGCAGAATTCA
GTGAATCATCGAGTCTTTGAACGCACATTGCGCCCCCTGGTATTCCGGGGG
GCATGCCTGTCCGAGCGTCATTGCTGCCCTCAAGCCCGGCTTGTGTGTTGG
GCCCTCGTCCCCCGGCTCCCGGGGGACGGGCCCGAAAGGCAGCGGCGGC
ACCGCGTCCGGTCCTCGAGCGTATGGGGCTTCGTCTTCCGCTCCGTAGGCC
CGGCCGGCGCCCGCCGACGCATTTATTTGCAACTTGTTTTTTTCCAGGTTGA
CCTCGGATCAGGTAGGGATACCCGCTGAACTTAAGCATATCAATAAGCCGG
GAGGAAG

The optical rotation values for compounds 1−18.

**1**	$[\alpha]_D^{25}$: −33.5 (*c* 0.39, MeOH)	**10**	$[\alpha]_D^{25}$: +129.3 (*c* 0.67, MeOH)
**2**	$[\alpha]_D^{25}$: −46.1 (*c* 0.43, MeOH)	**11**	$[\alpha]_D^{25}$: +89.1 (*c* 0.24, MeOH)
**3**	$[\alpha]_D^{25}$: −31.1 (*c* 0.33, MeOH)	**12**	$[\alpha]_D^{25}$: +31.3 (*c* 0.17, MeOH)
**4**	$[\alpha]_D^{25}$: +156.1 (*c* 0.15, MeOH)	**13**	$[\alpha]_D^{25}$: +112.3 (*c* 0.35, MeOH)
**5**	$[\alpha]_D^{25}$: +179.5 (*c* 0.16, MeOH)	**14**	$[\alpha]_D^{25}$: +31.2 (*c* 0.21, MeOH)
**6**	$[\alpha]_D^{25}$: 0 (*c* 0.15, MeOH)<br>Plane structure without optical rotation value	**15**	$[\alpha]_D^{25}$: +56.8 (*c* 0.18, MeOH)
**7**	$[\alpha]_D^{25}$: +17.9 (*c* 0.13, MeOH)	**16**	$[\alpha]_D^{25}$: +21.1 (*c* 0.12, MeOH)
**8**	$[\alpha]_D^{25}$: +115.3 (*c* 0.51, MeOH)	**17**	$[\alpha]_D^{25}$: +51.7 (*c* 0.24, MeOH)
**9**	$[\alpha]_D^{25}$: +25.1 (*c* 0.17, MeOH)	**18**	$[\alpha]_D^{25}$: +112.3 (*c* 0.81, MeOH)

Figure S1. (A) Position of AH (left) and PLP (right) into the active site of GOT1. (B) Surface representation of GOT1 around the active site pocket. (C) Overlay of GOT1-AH (cyan) and WT GOT1 (magenta) crystal structures. (D) Comparison of the center conformations of core molecules in chain A of GOT1-AH (cyan) and wild-type GOT1 (gray).

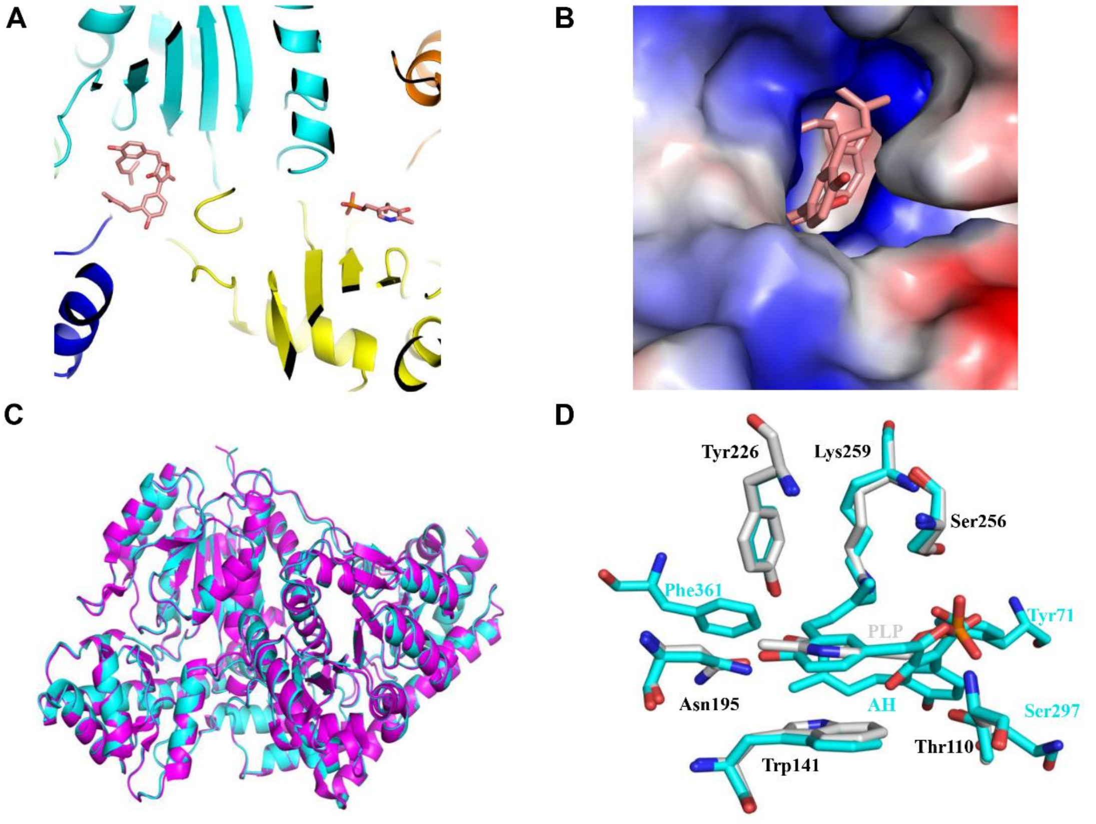

Figure S2. The 2Fo-Fc electron density of PLP bound to chain B is shown as a gray mesh. The map is contoured at the 1σ level.

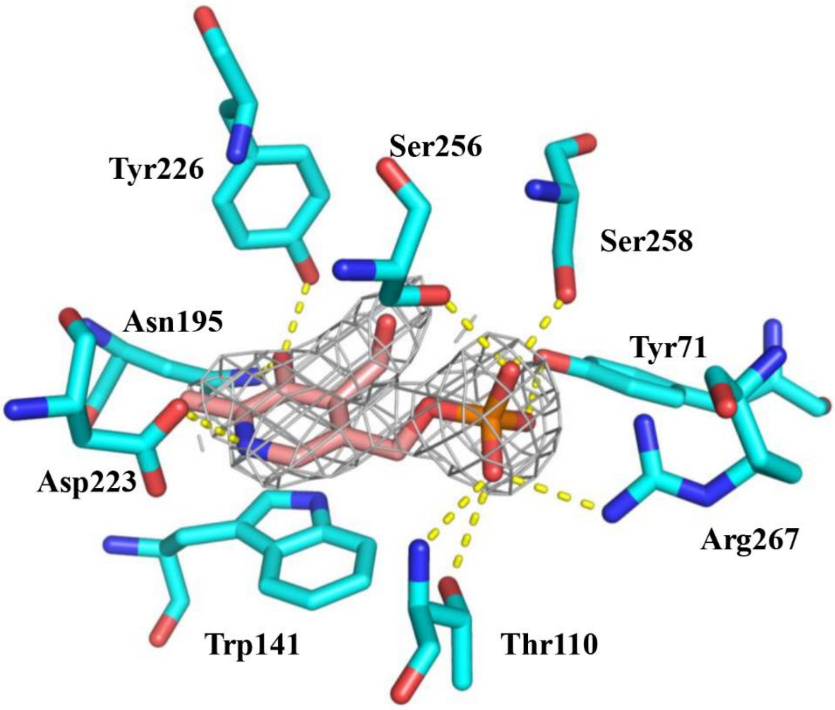

Table S1. Data collection and refinement statistics.

parameter	Enzyme in complex with AH
Resolution (Å)	49.58–2.62 (2.714–2.62)
Space group	$P\ 1\ 2_1\ 1$
Unit cell dimensions	a=64.87 Å, b=90.42 Å, c=74.05 Å;
	α=90.0º, β =91.87º, γ=90.0º
Unique reflections	25267 (2557)
Multiplicity	2.0 (2.0)
Completeness (%)	97.67 (97.93)
$I/\sigma(I)$	9.34 (2.99)
R-merge† (%)	0.07031 (0.2735)
R-work	0.2127 (0.2690)
R-free	0.2661 (0.3291)
Number of non-hydrogen atoms	6,703
Protein residues	820
r.m.s.d.from ideal geometry	
Bonds lengths (Å)	0.002
Angles (º)	0.46
Ramachandran plot (%)	
Favored	96.94
Outliers	0.12
Clashscore	4.51
Average *B*-factor	29.95
PDB code	6LIG

†*R*-merge = $\Sigma_{hkl} \Sigma_i |I_i(hkl) - \langle I(hkl)\rangle| / \Sigma_{hkl} \Sigma_i I_i(hkl)$, where $I(hkl)$ is the intensity of reflection *hkl*, Σ_{hkl} is the sum over all reflections and Σ_i is the sum over *i* measurements of reflection *hkl*. Statistics for the highest-resolution shell are shown in parentheses.

Figure S3. ^{1}H NMR spectrum of **1**.

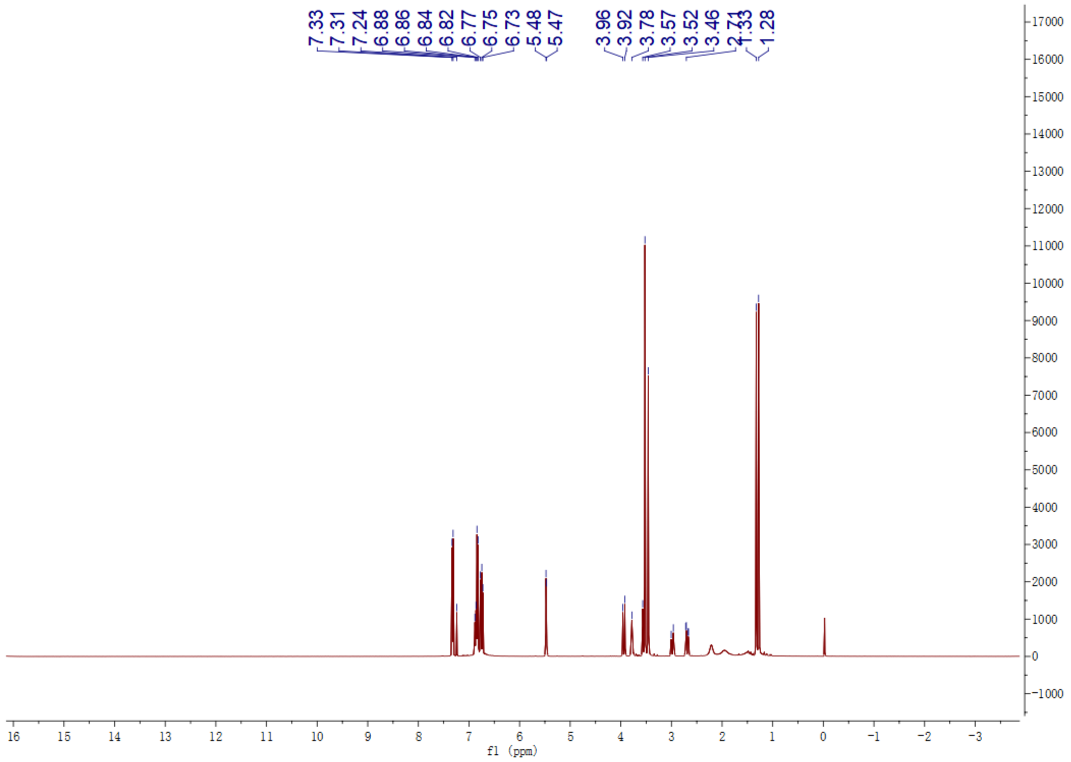

Figure S4. ^{13}C NMR spectrum of **1**.

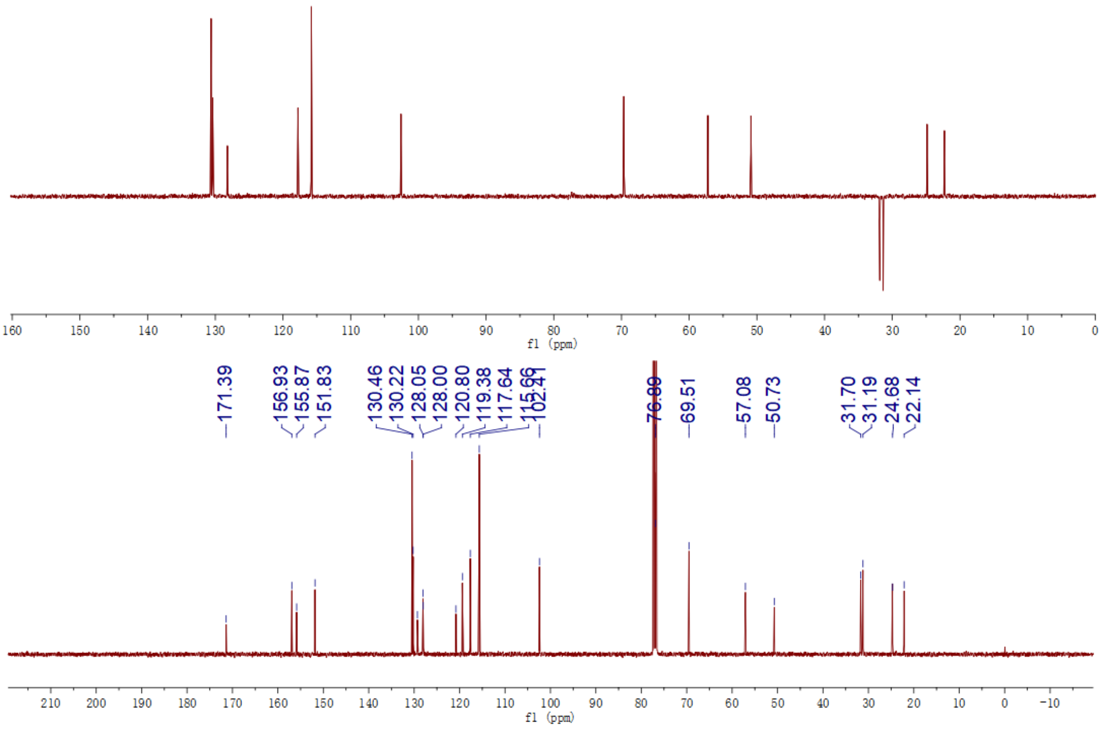

Figure S5. ^{1}H NMR spectrum of **2**.

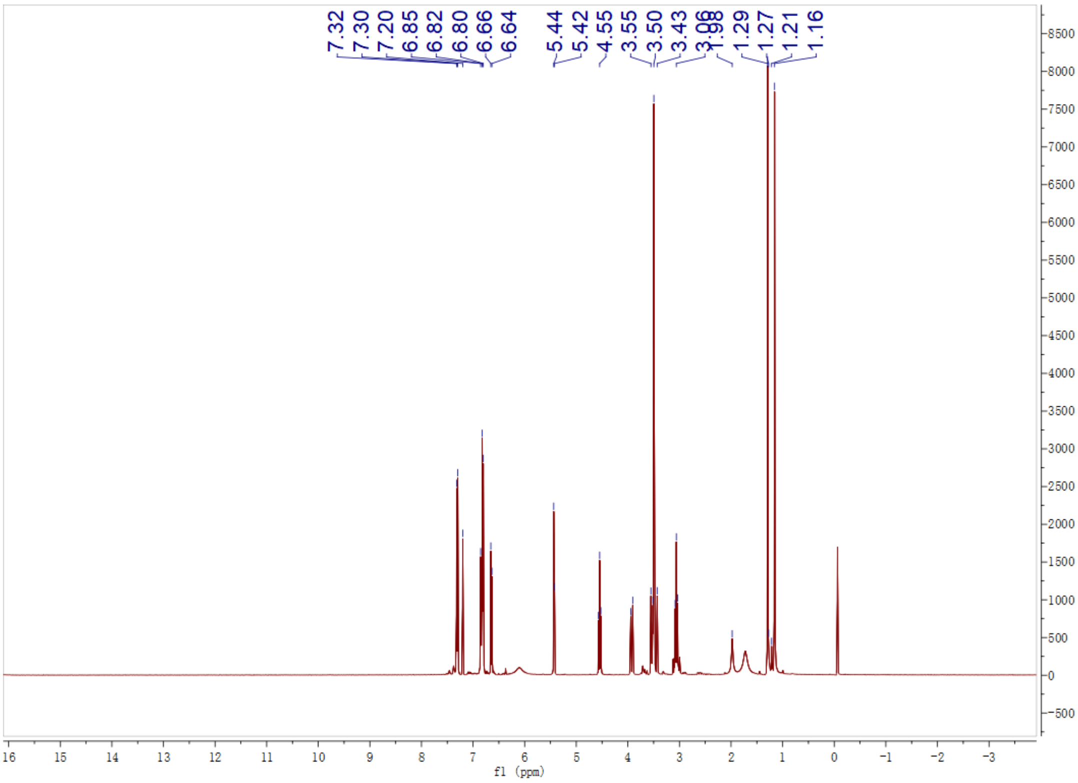

Figure S6. ^{13}C NMR spectrum of **2**.

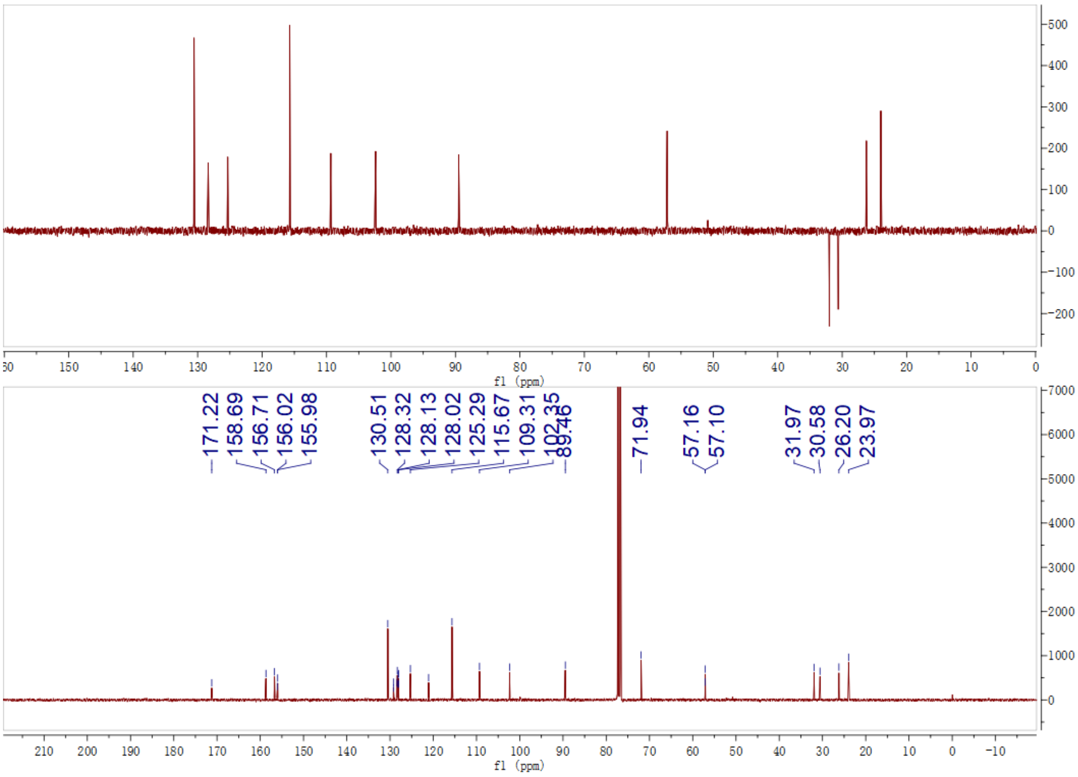

Figure S7. ^{1}H NMR spectrum of **3**.

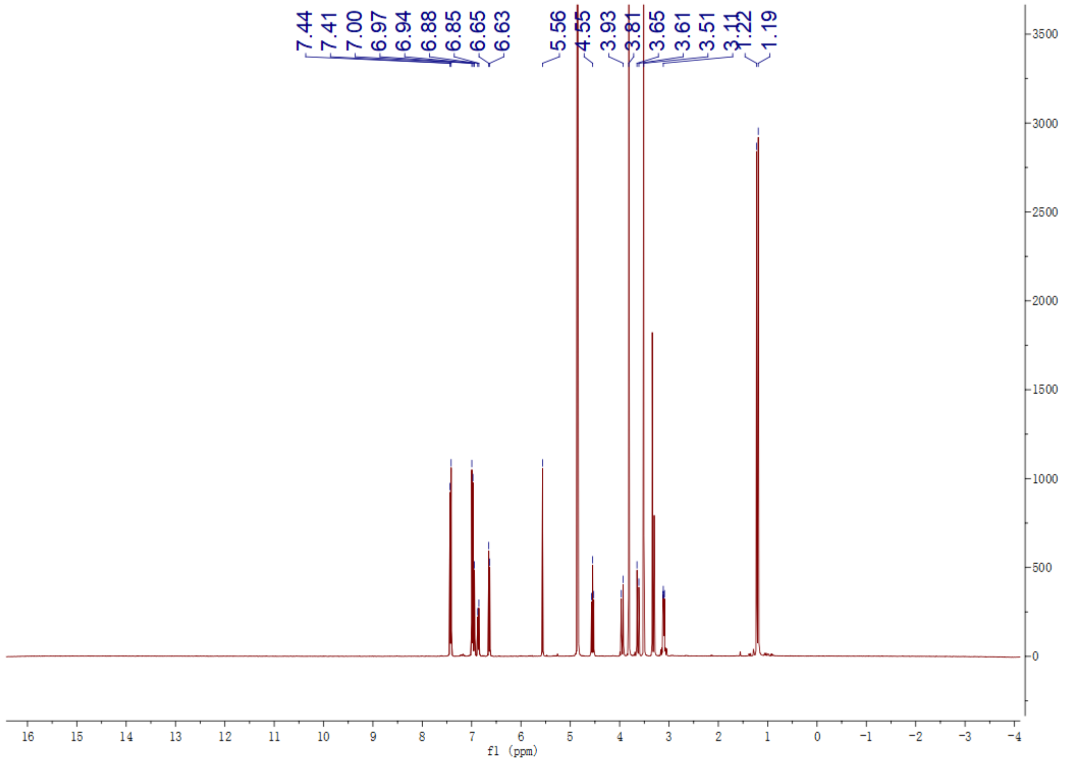

Figure S8. ^{13}C NMR spectrum of **3**.

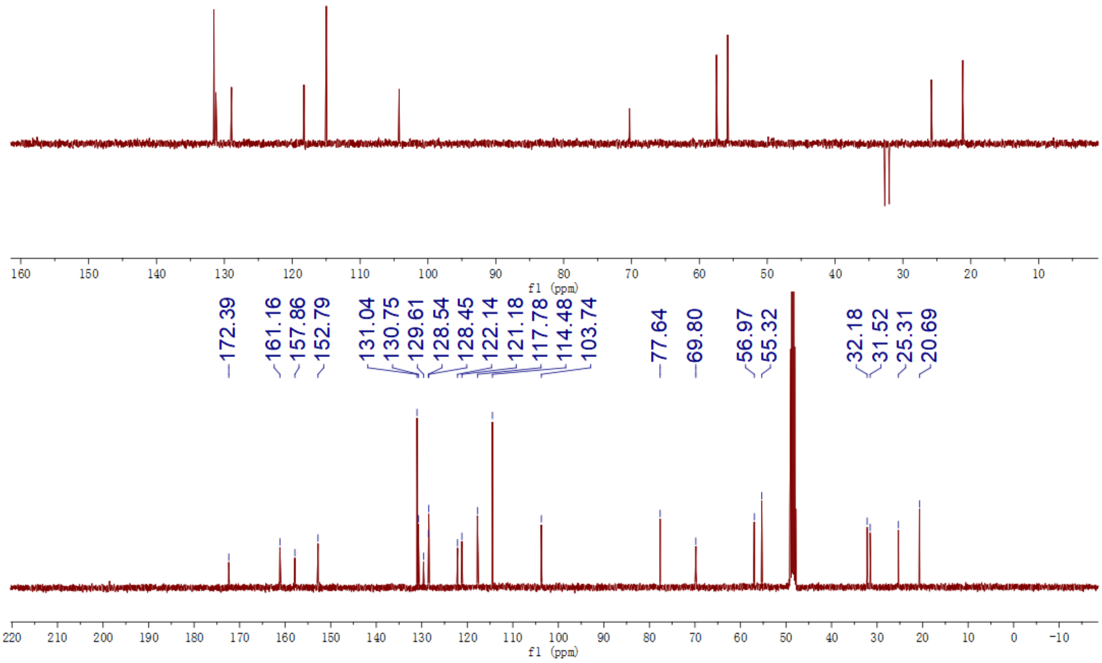

Figure S9. ^{1}H NMR spectrum of **4**.

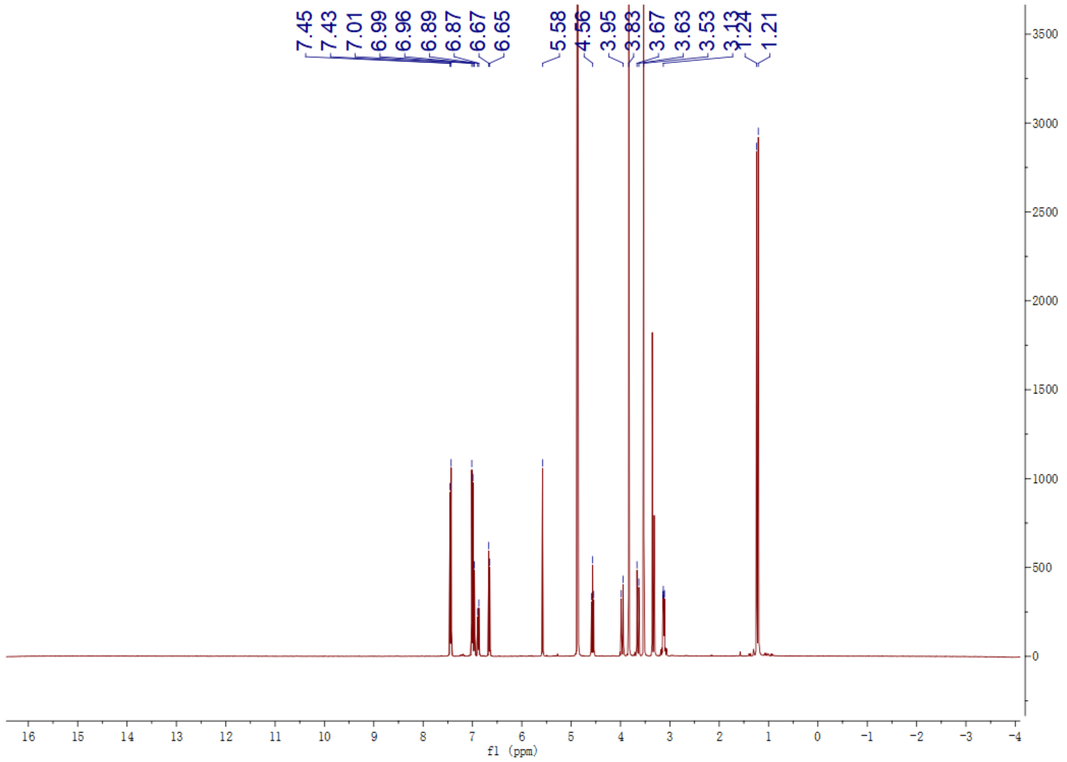

Figure S10. ^{13}C NMR spectrum of **4**.

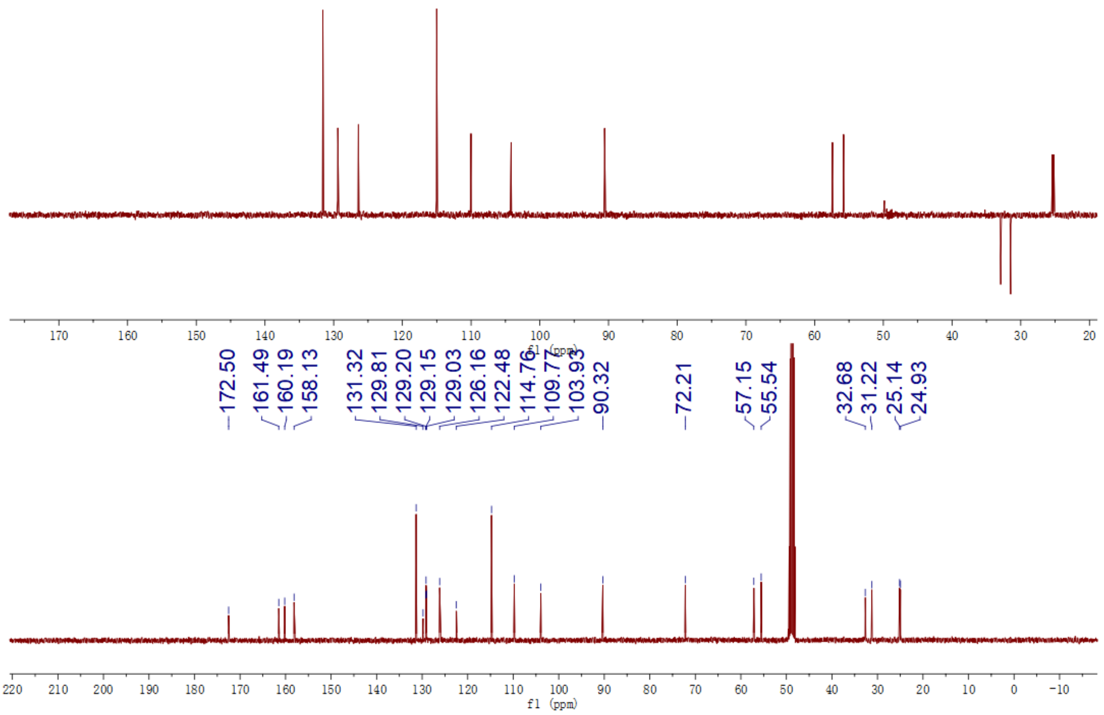

Figure S11. ^{1}H NMR spectrum of **5**.

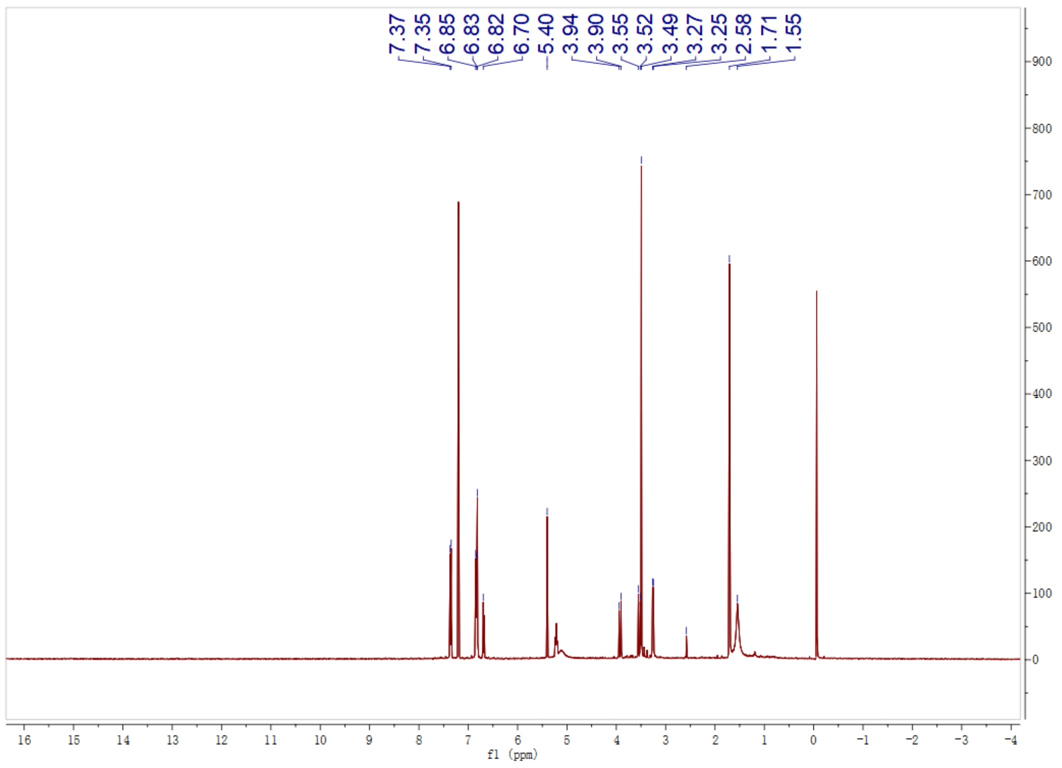

Figure S12. ^{13}C NMR spectrum of **5**.

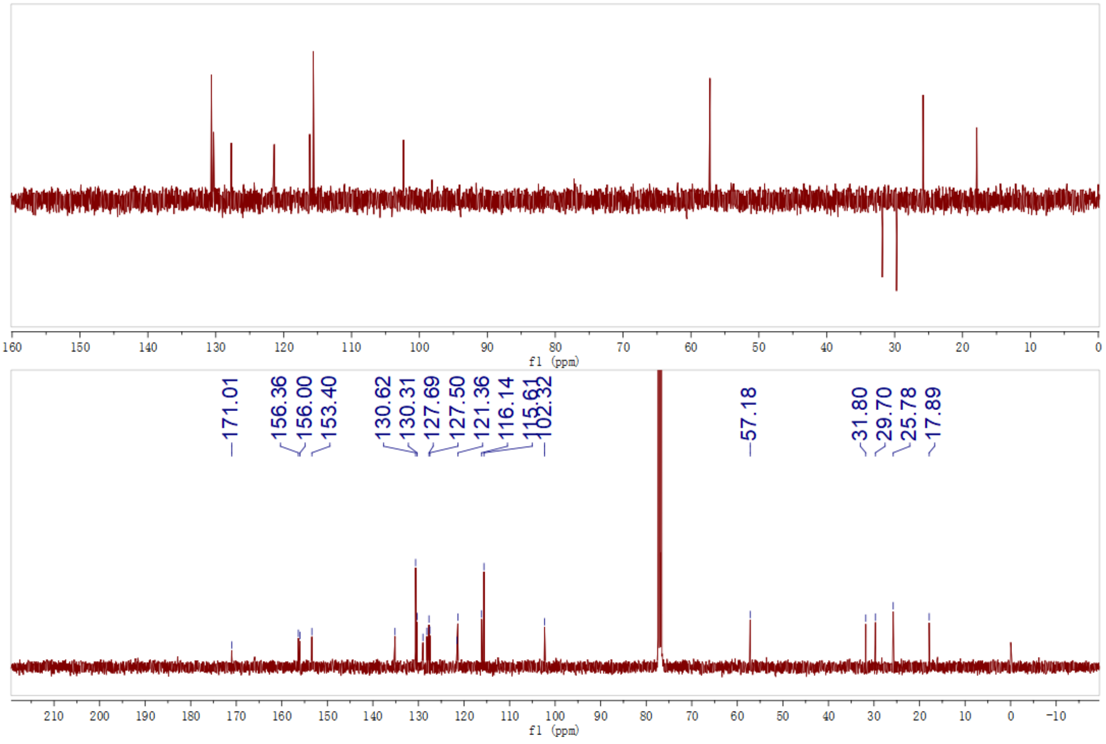

Figure S13. ^{1}H NMR spectrum of **6**.

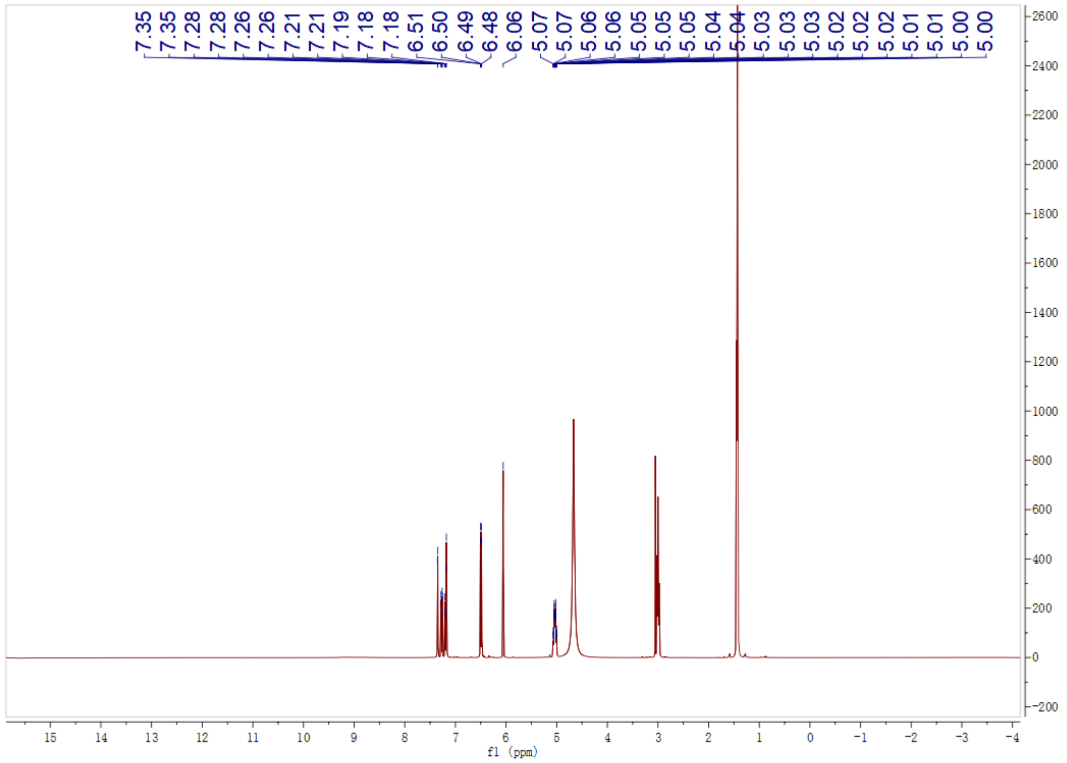

Figure S14. ^{13}C NMR spectrum of **6**.

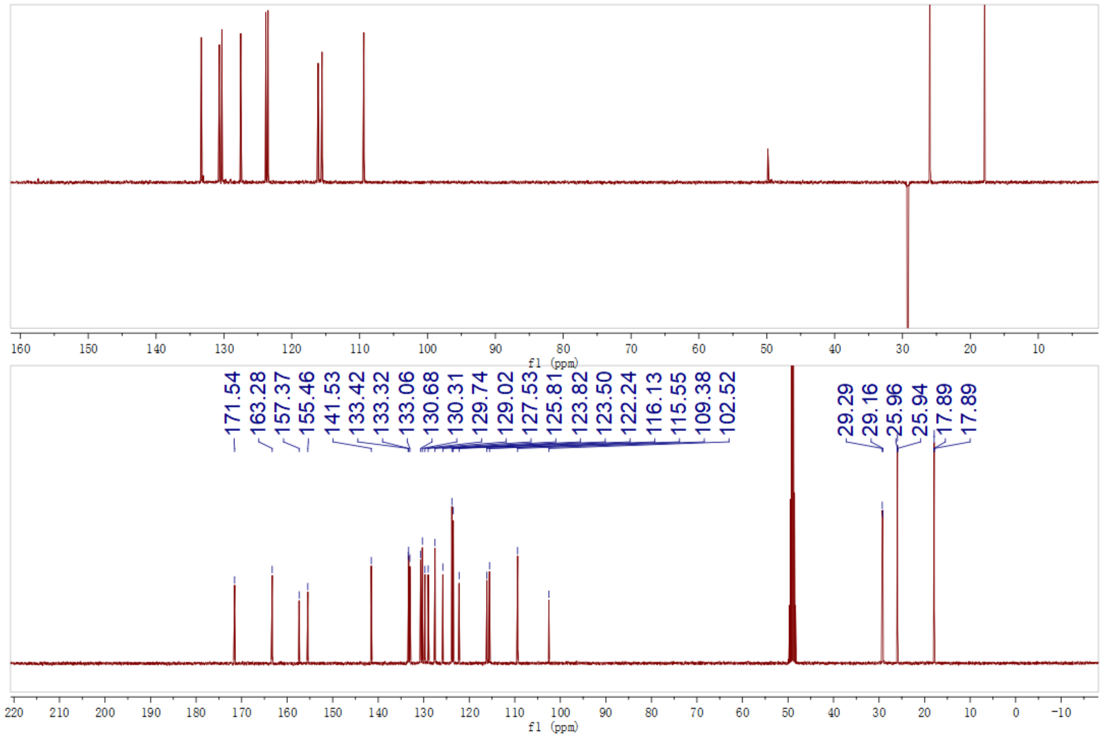

Figure S15. ^{1}H NMR spectrum of **7**.

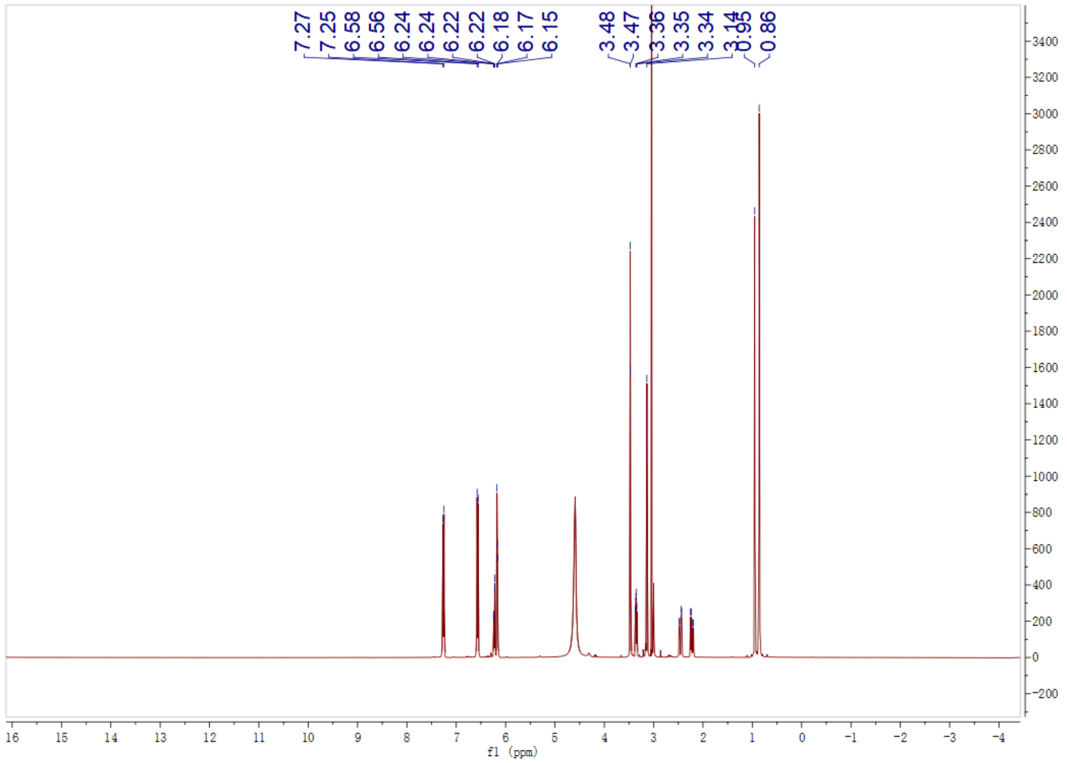

Figure S16. ^{13}C NMR spectrum of **7**.

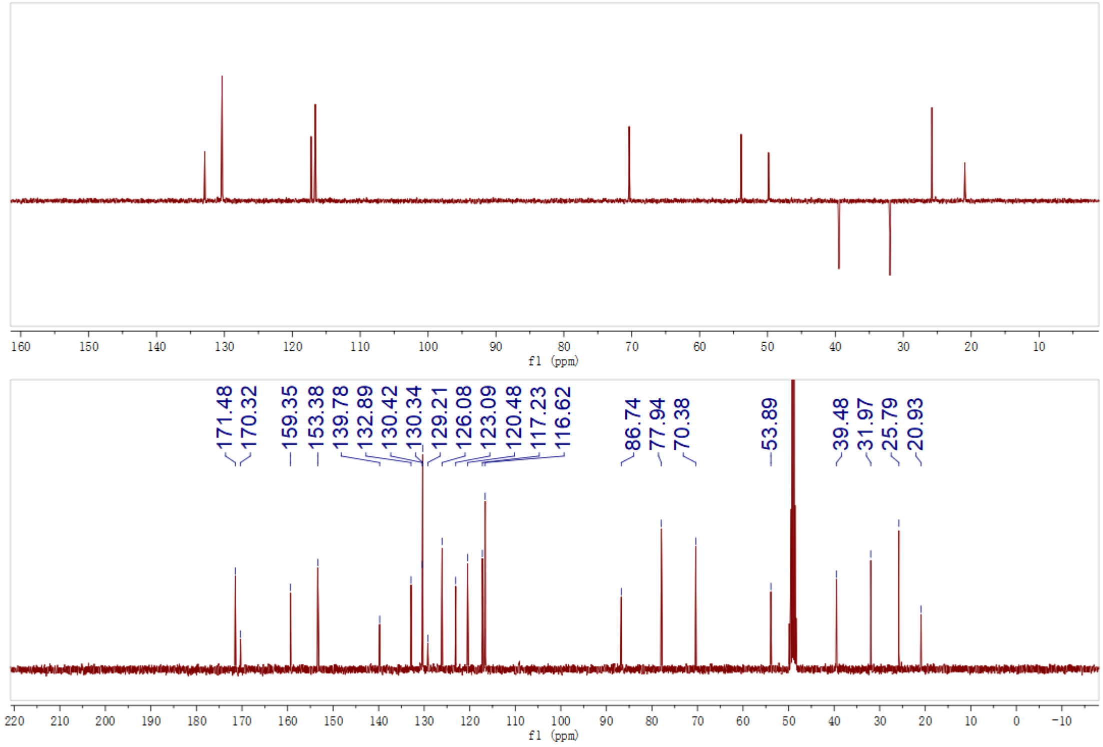

Figure S17. ^{1}H NMR spectrum of **8**.

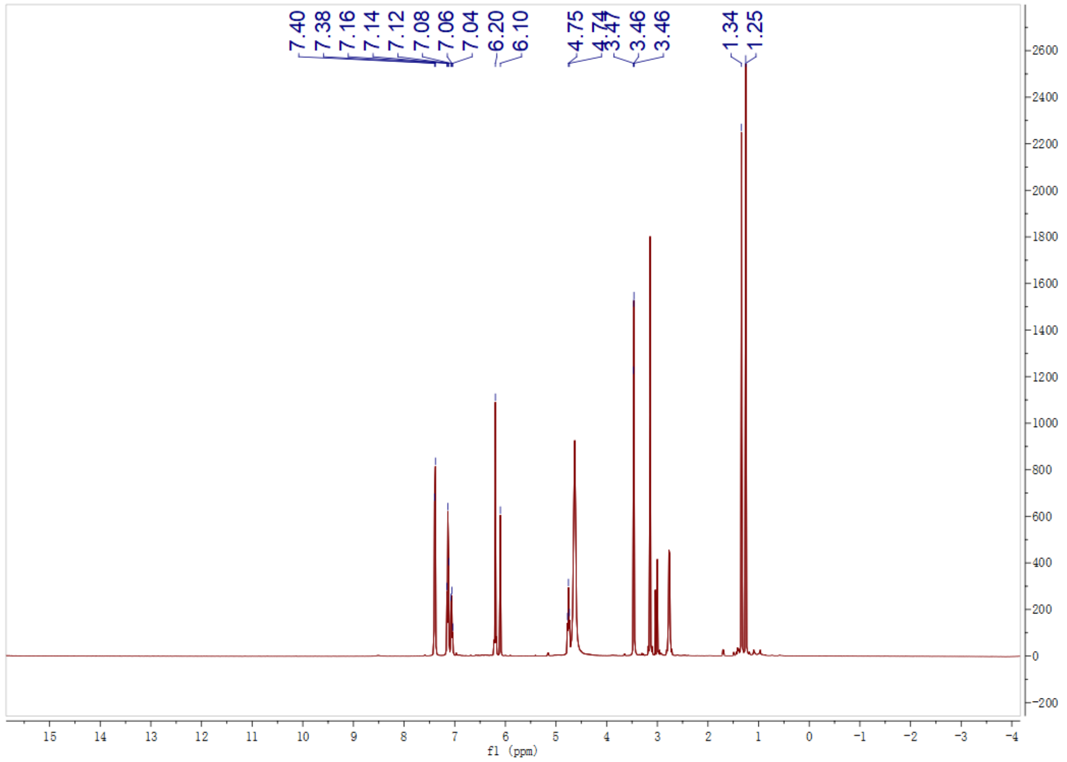

Figure S18. ^{13}C NMR spectrum of **8**.

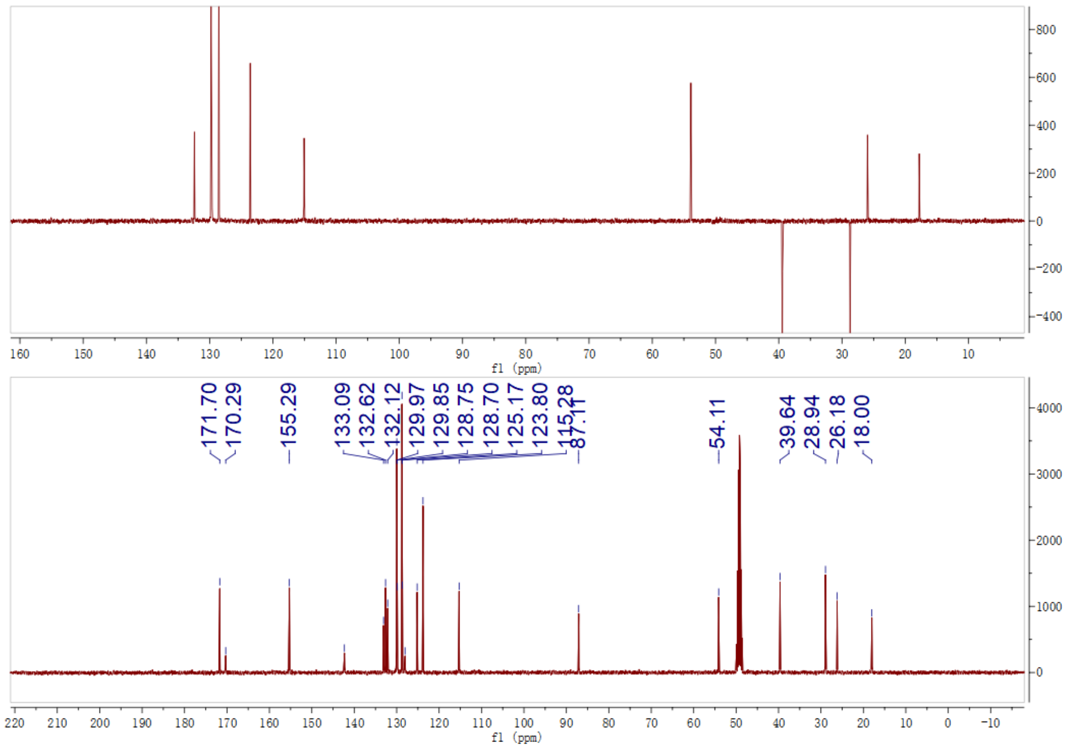

Figure S19. ^{1}H NMR spectrum of **9**.

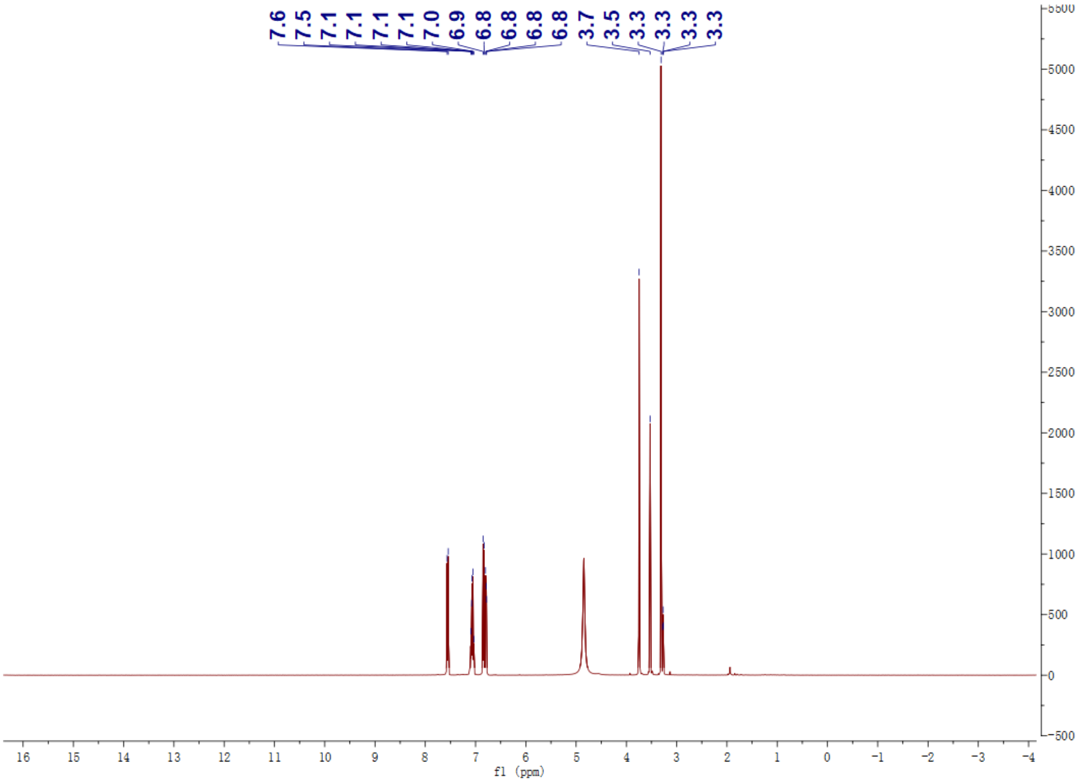

Figure S20. ^{13}C NMR spectrum of **9**.

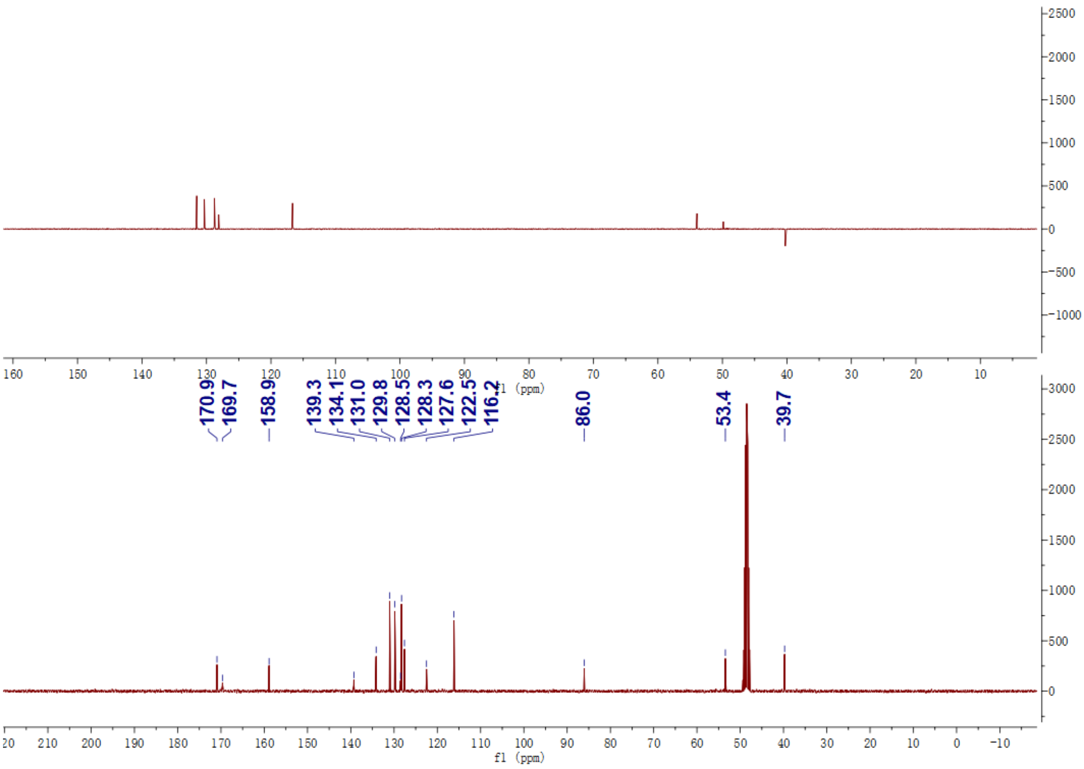

Figure S21. ^{1}H NMR spectrum of **10**.

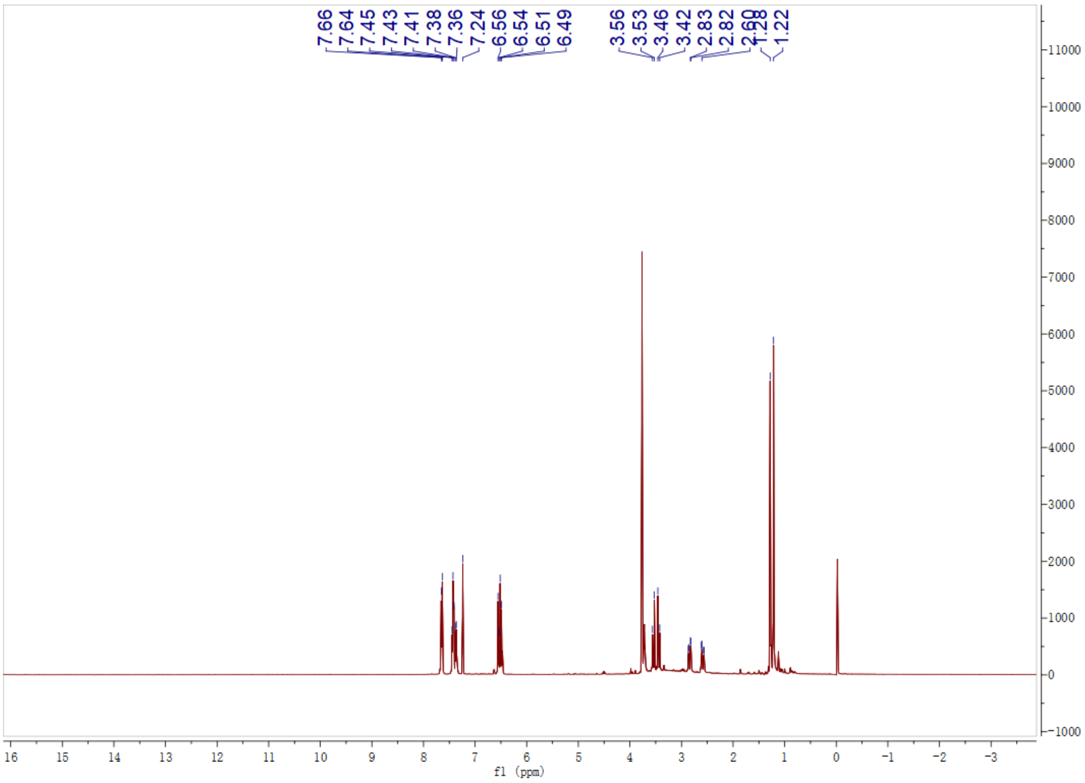

Figure S22. ^{13}C NMR spectrum of **10**.

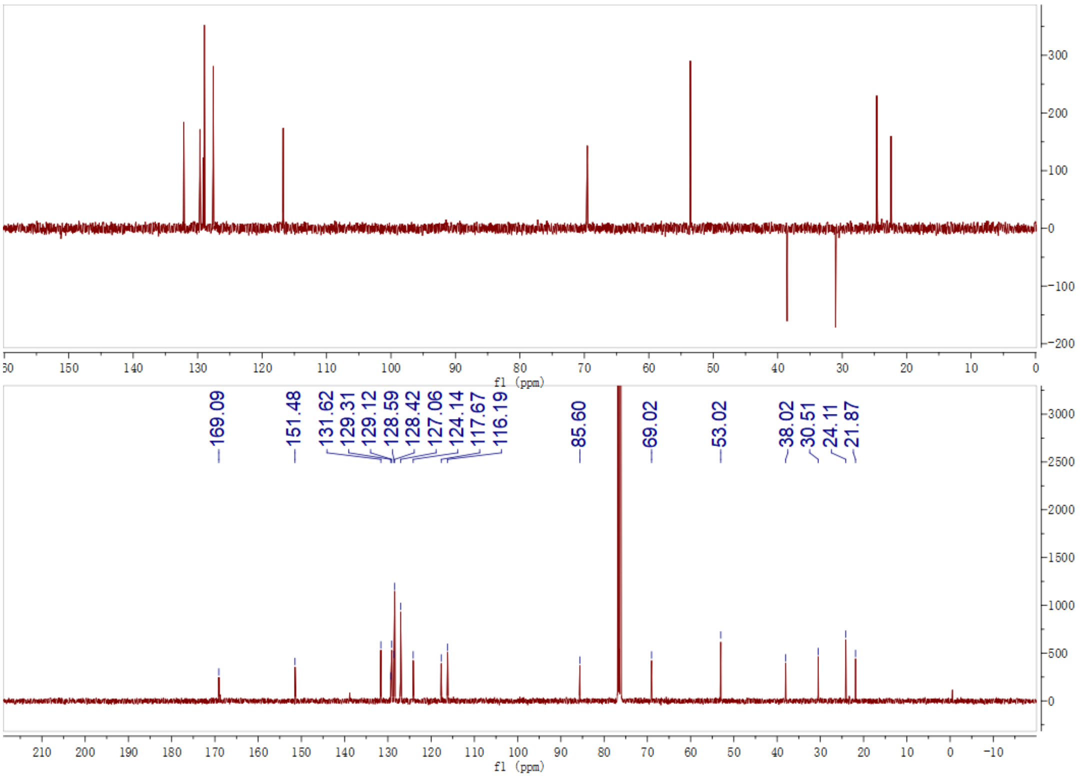

Figure S23. ^{1}H NMR spectrum of **11**.

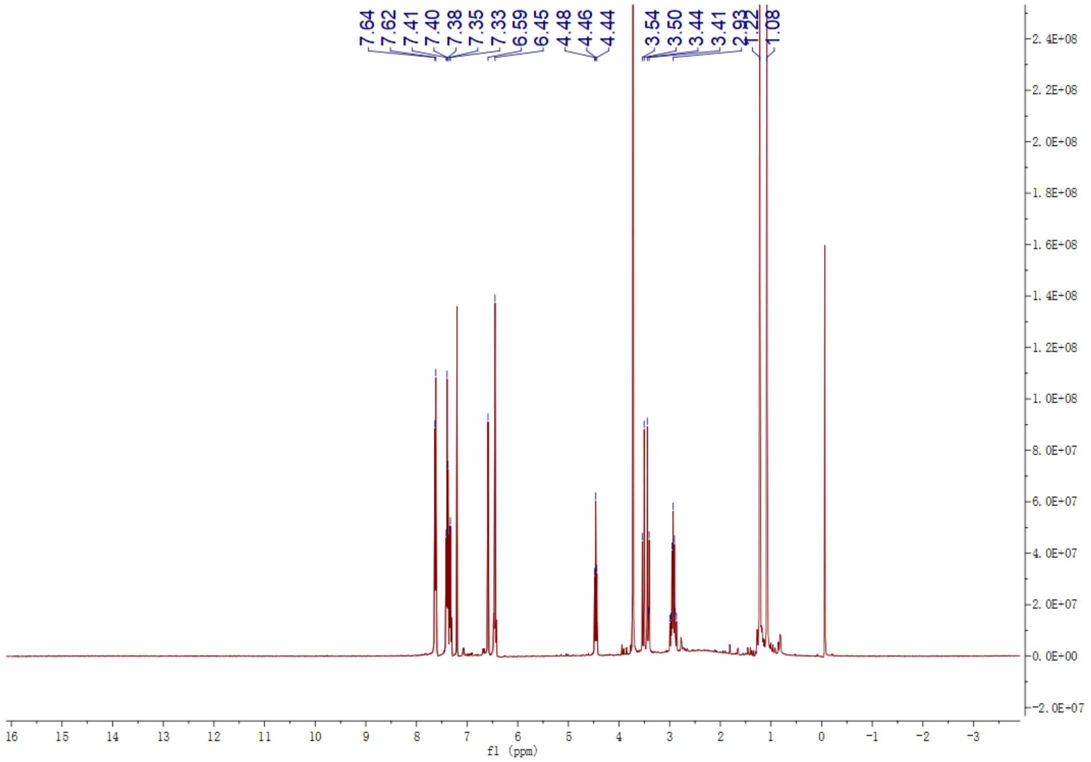

Figure S24. ^{13}C NMR spectrum of **11**.

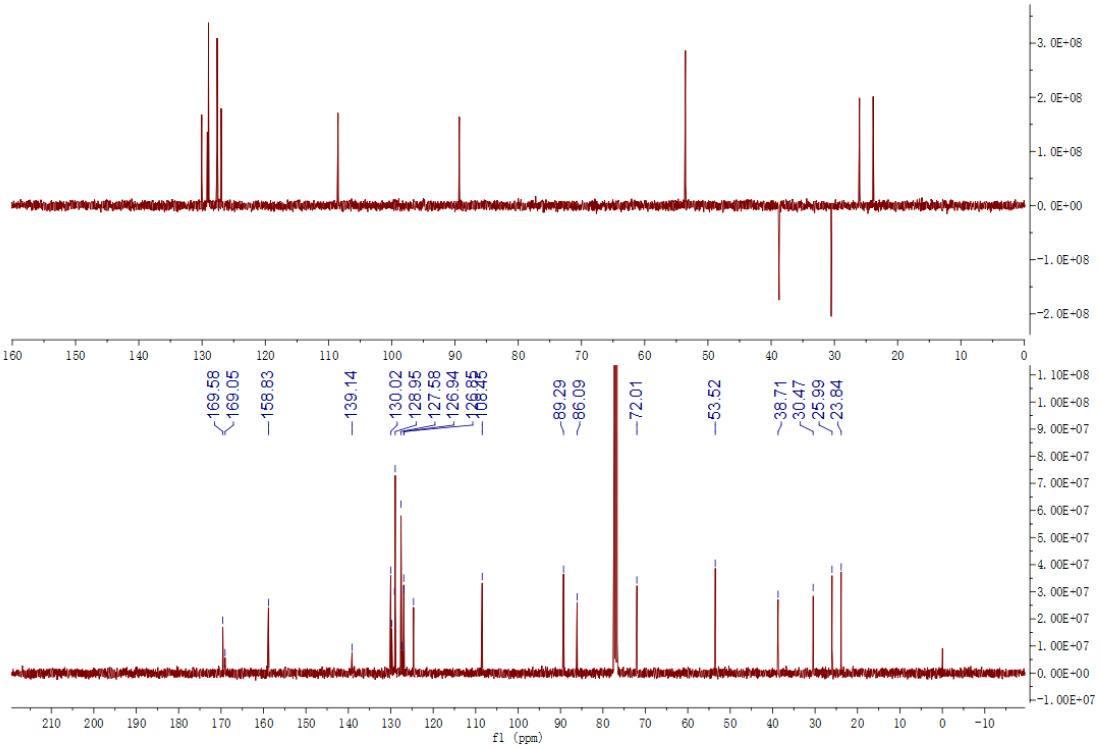

Figure S25. ^{1}H NMR spectrum of **12**.

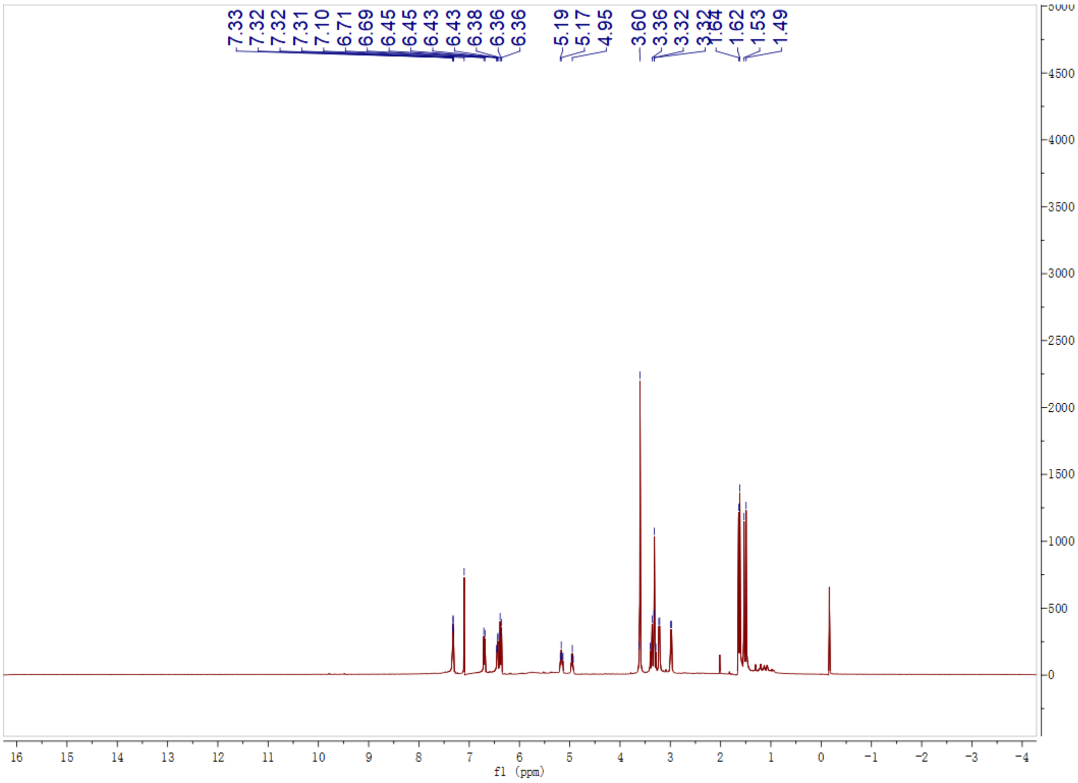

Figure S26. ^{13}C NMR spectrum of **12**.

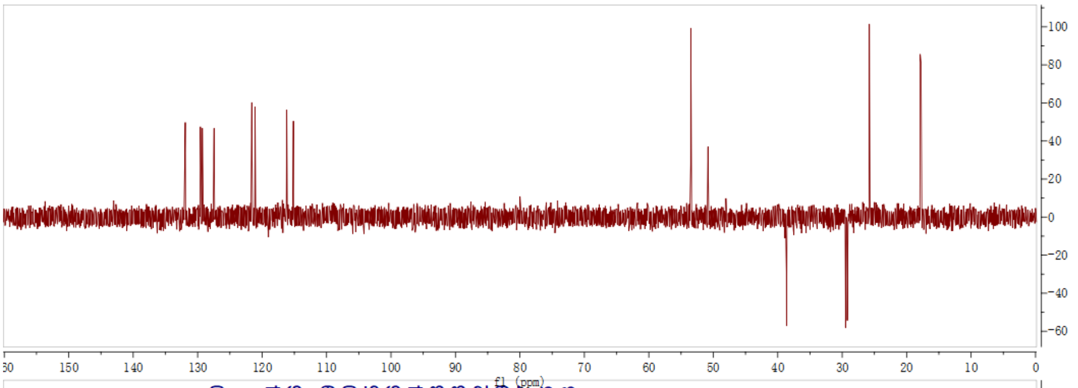

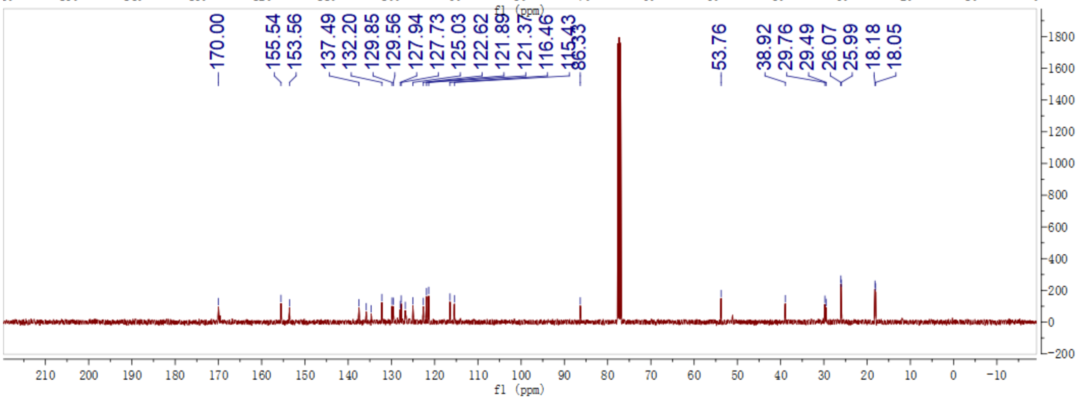

Figure S27. ^{1}H NMR spectrum of **13**.

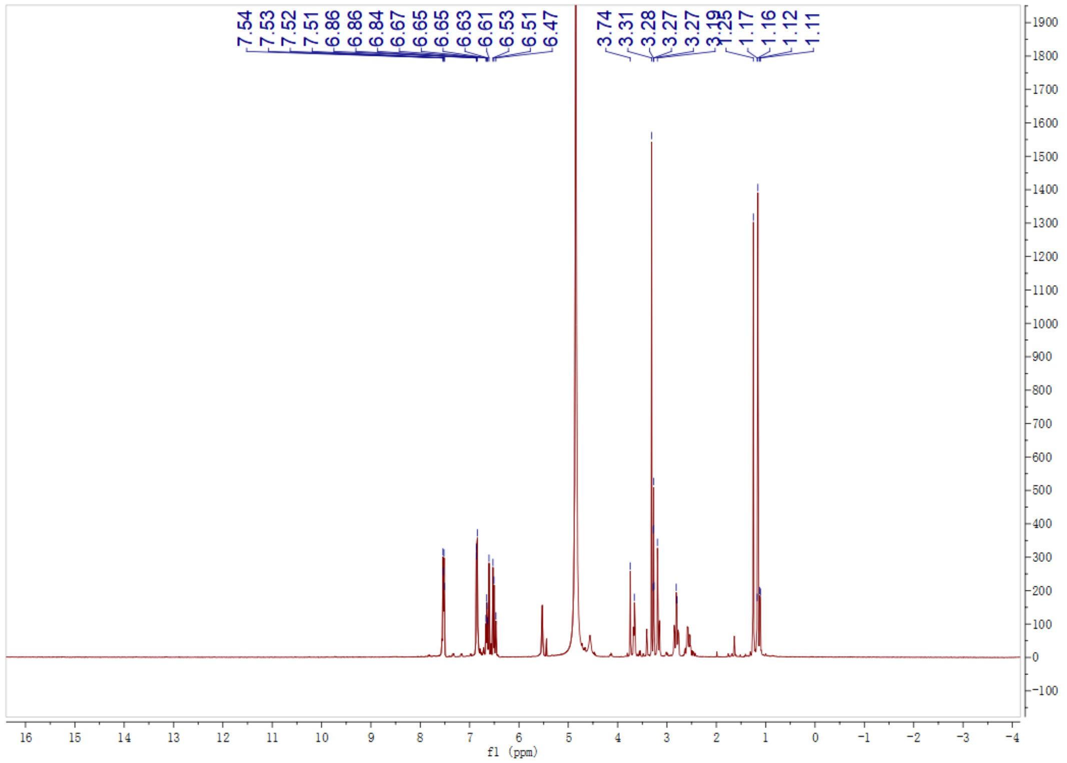

Figure S28. ^{13}C NMR spectrum of **13**.

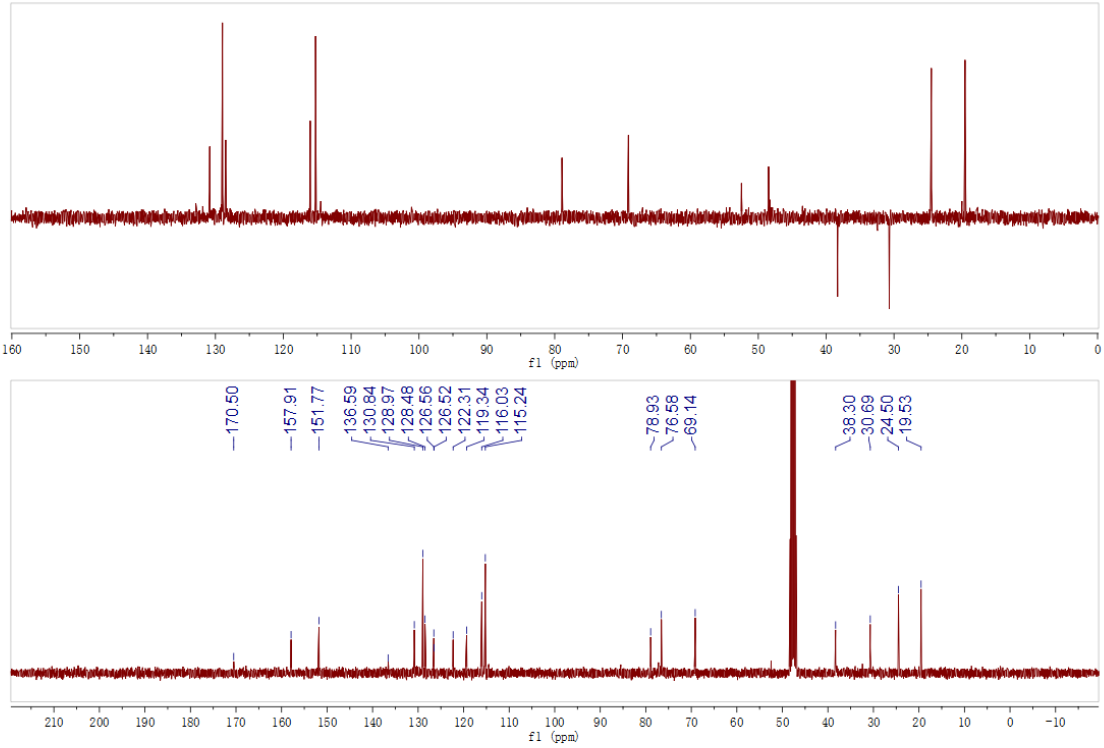

Figure S29. ^{1}H NMR spectrum of **14**.

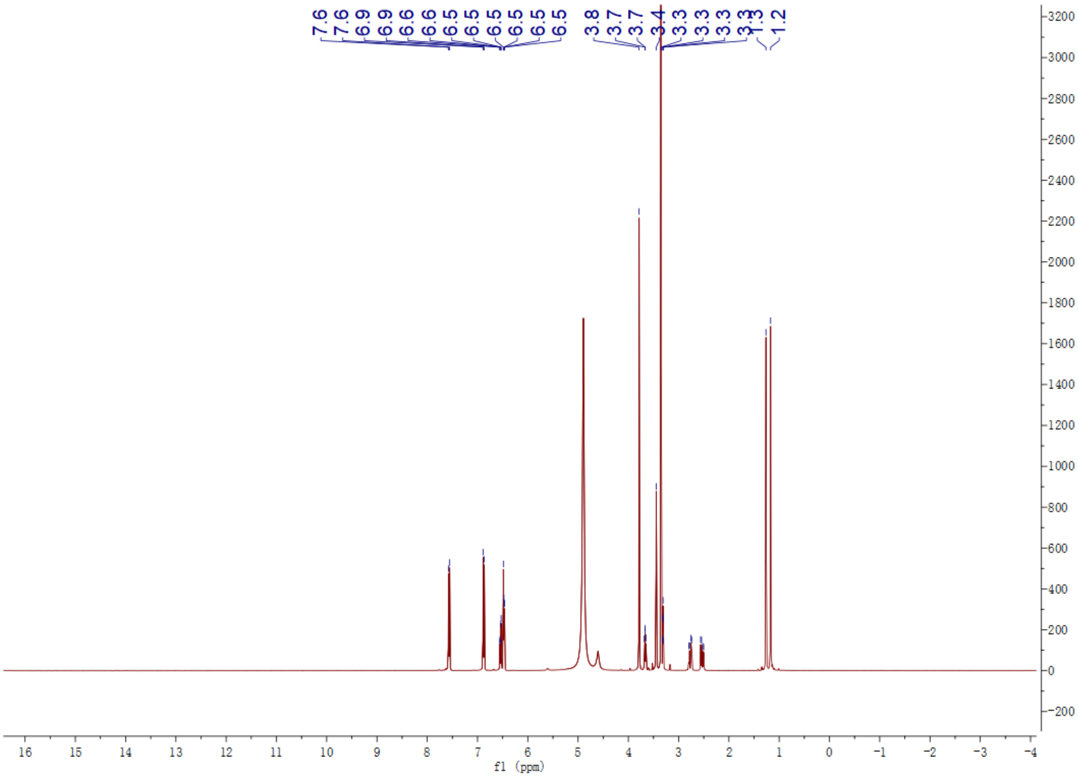

Figure S30. ^{13}C NMR spectrum of **14**.

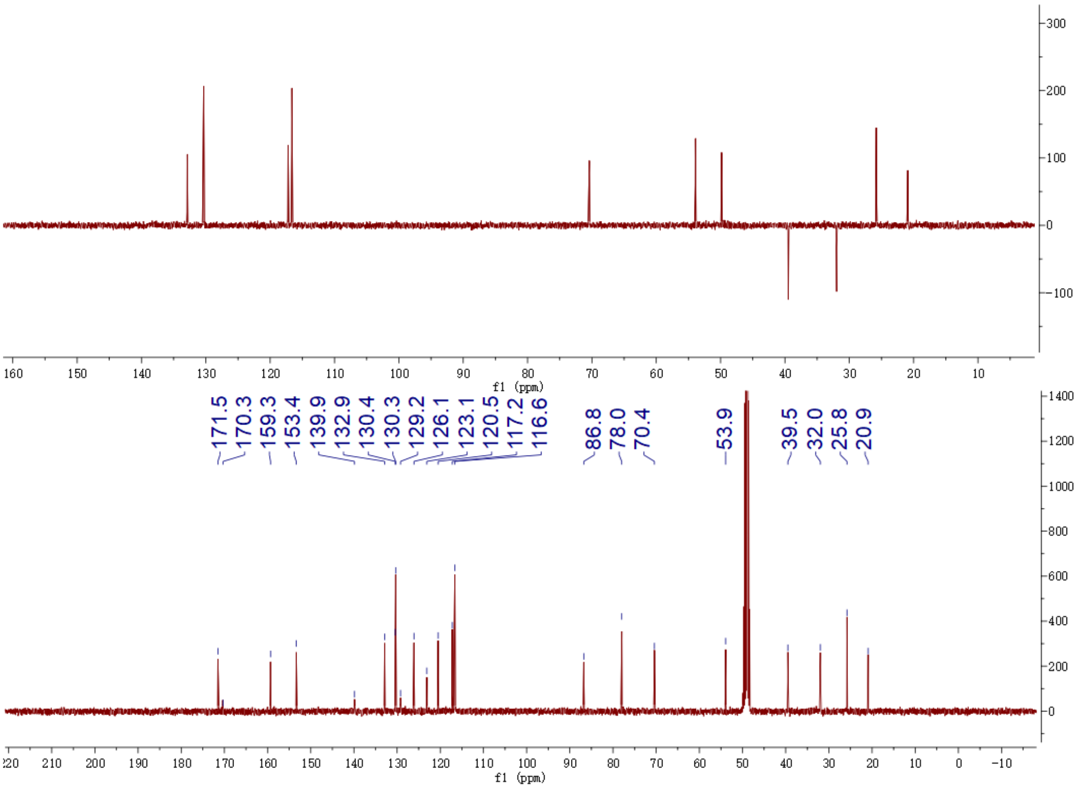

Figure S31. ^{1}H NMR spectrum of **15**.

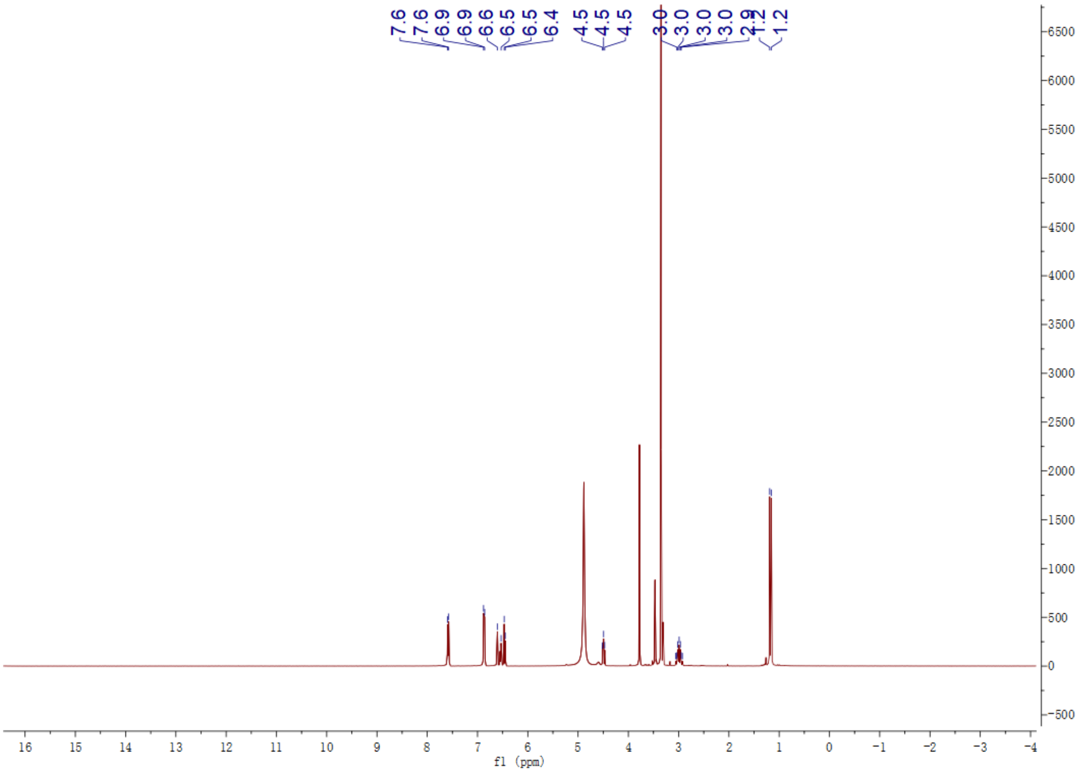

Figure S32. ^{13}C NMR spectrum of **15**.

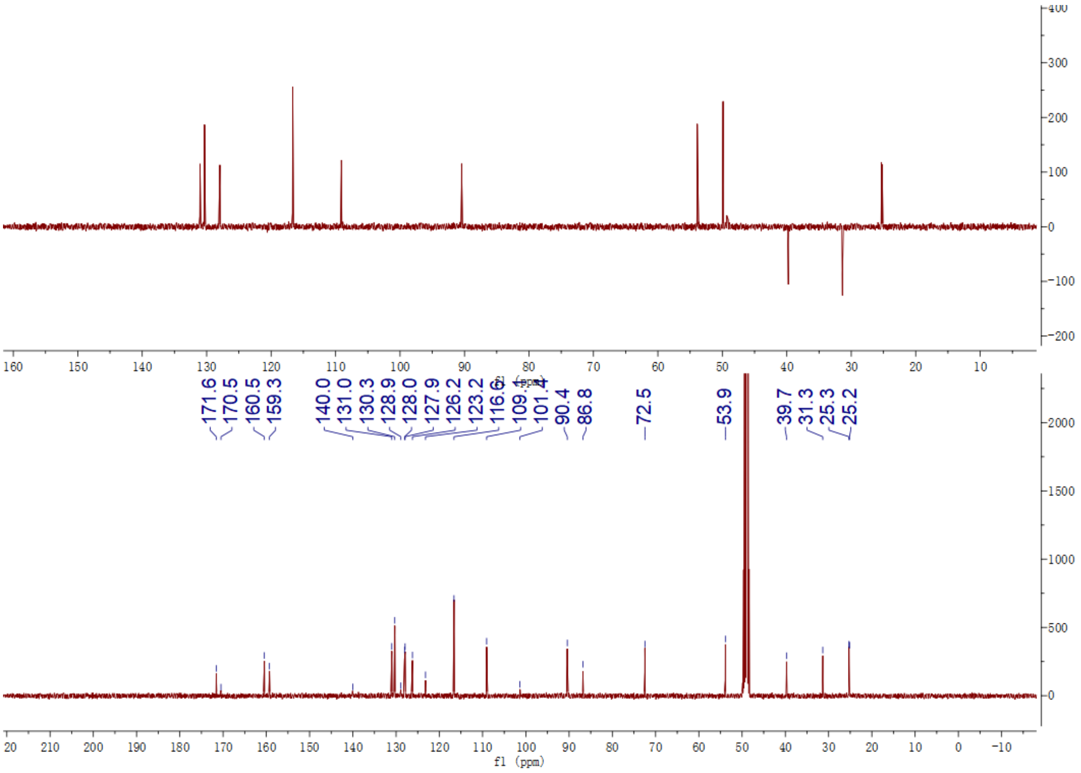

Figure S33. ^{1}H NMR spectrum of **16**.

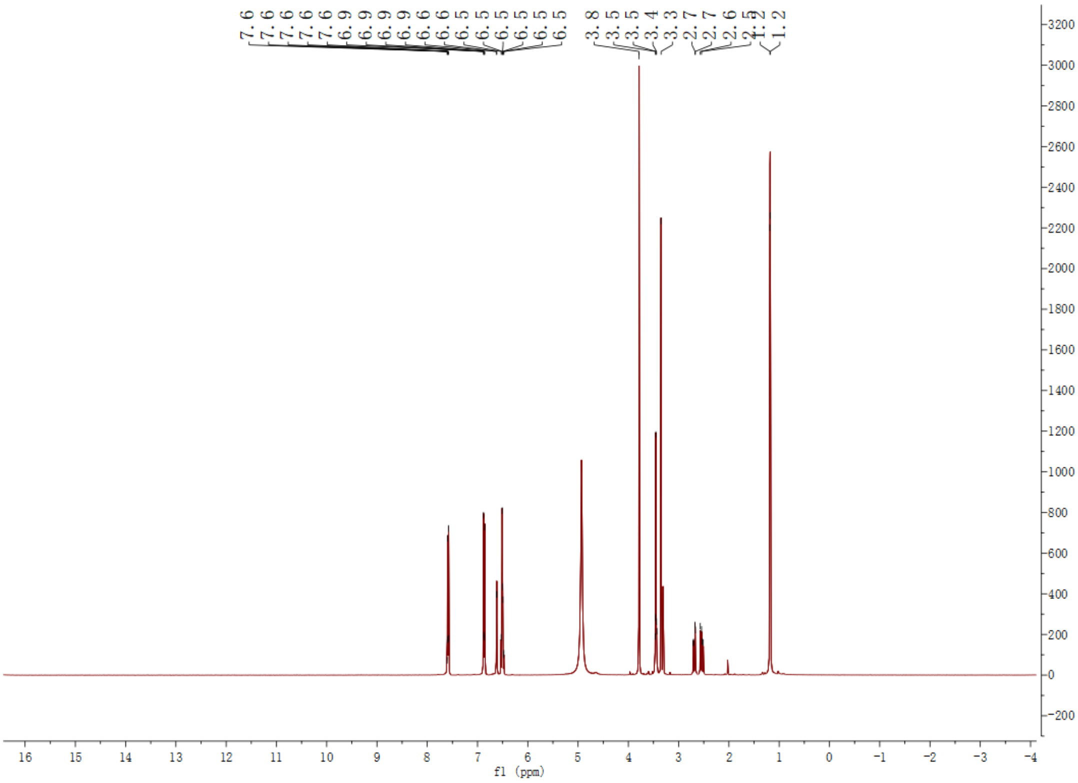

Figure S34. ^{13}C NMR spectrum of **16**.

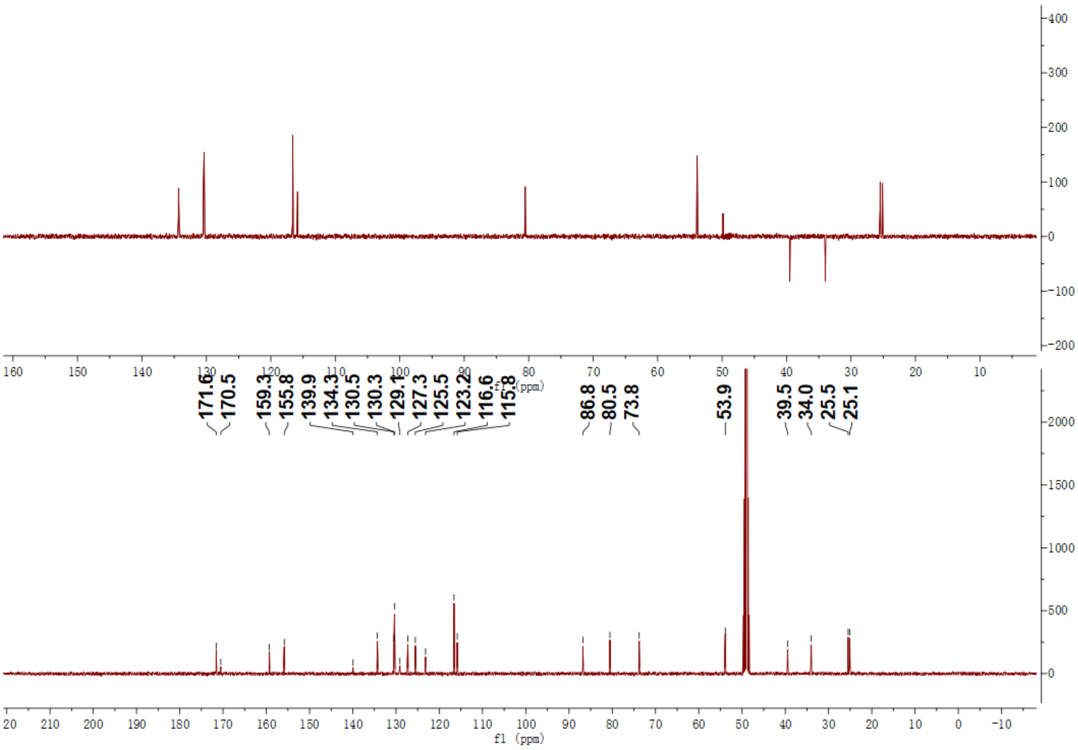

Figure S35. ^{13}C NMR spectrum of **17**.

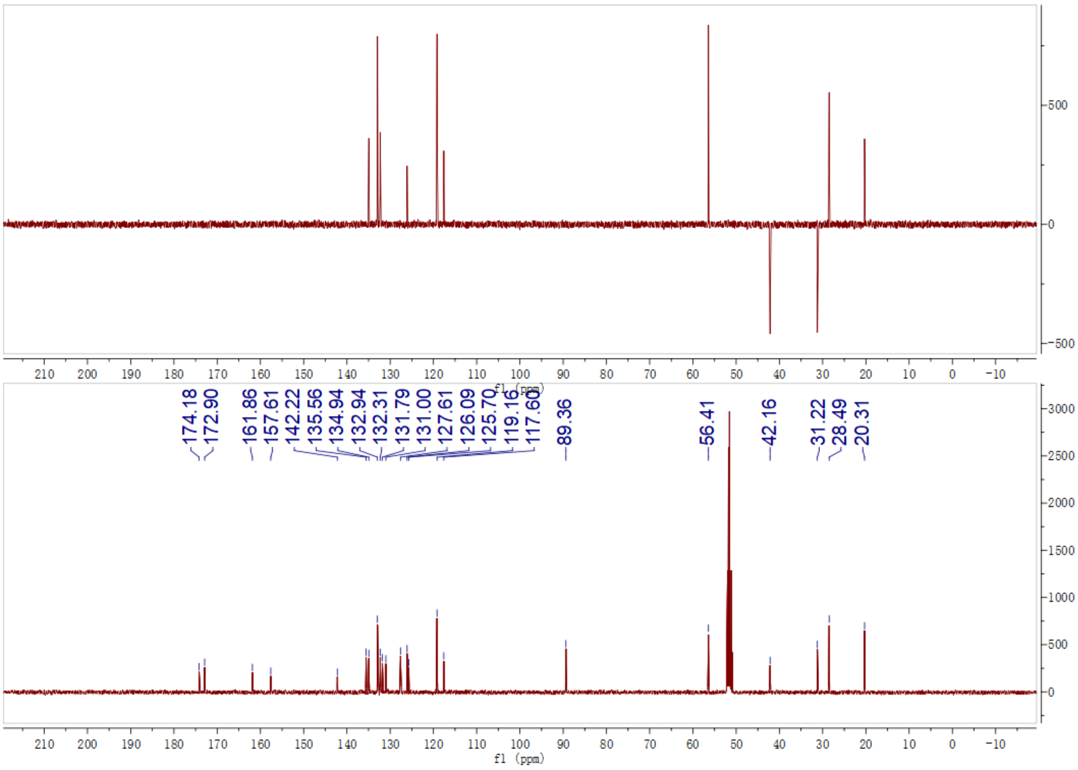

Figure S36. ^{1}H NMR spectrum of **18**.

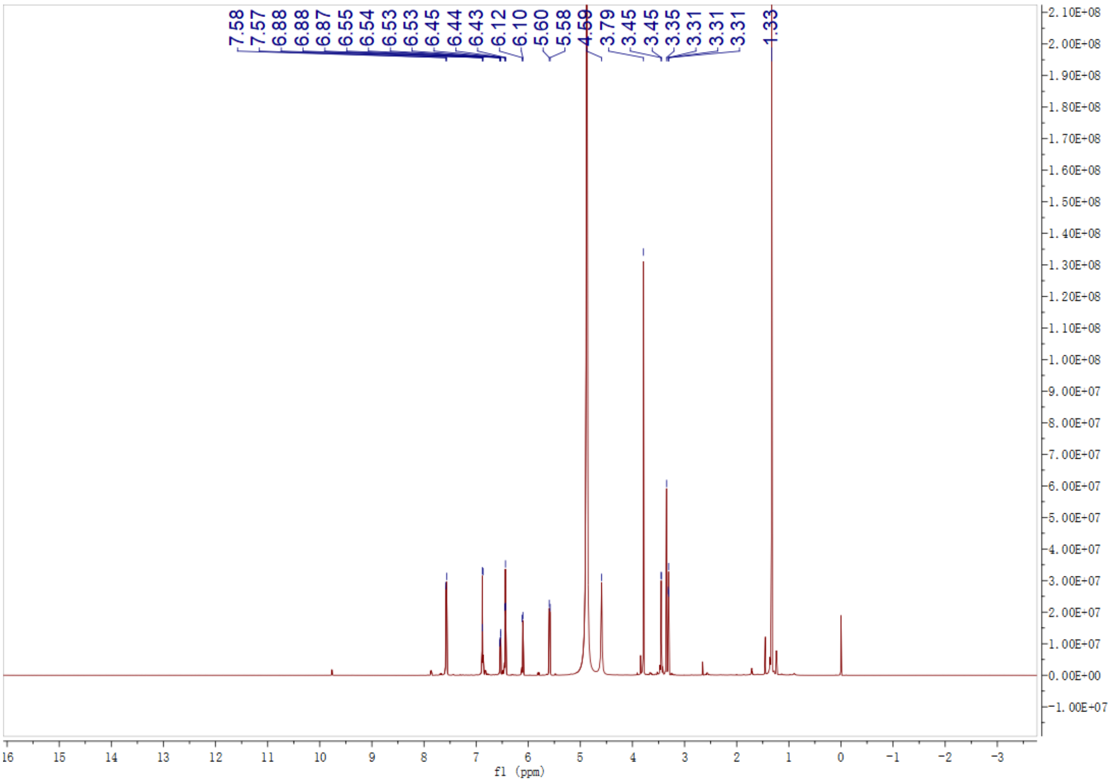

Figure S37. ^{13}C NMR spectrum of **18**.

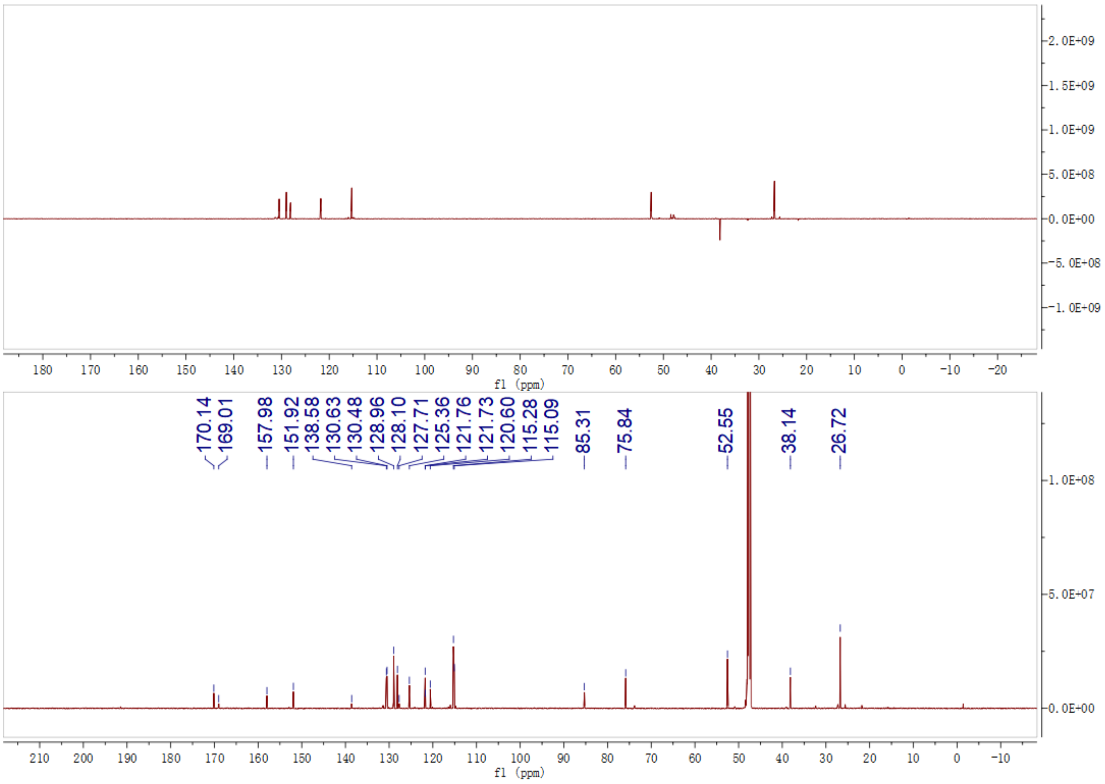

Figure S38. HPLC profile for the extract of the culture of *Aspergillus terreus* [MeOH–H_2O (20:80 to 80:20, 80 min, v/v, 2.0 mL/min)].

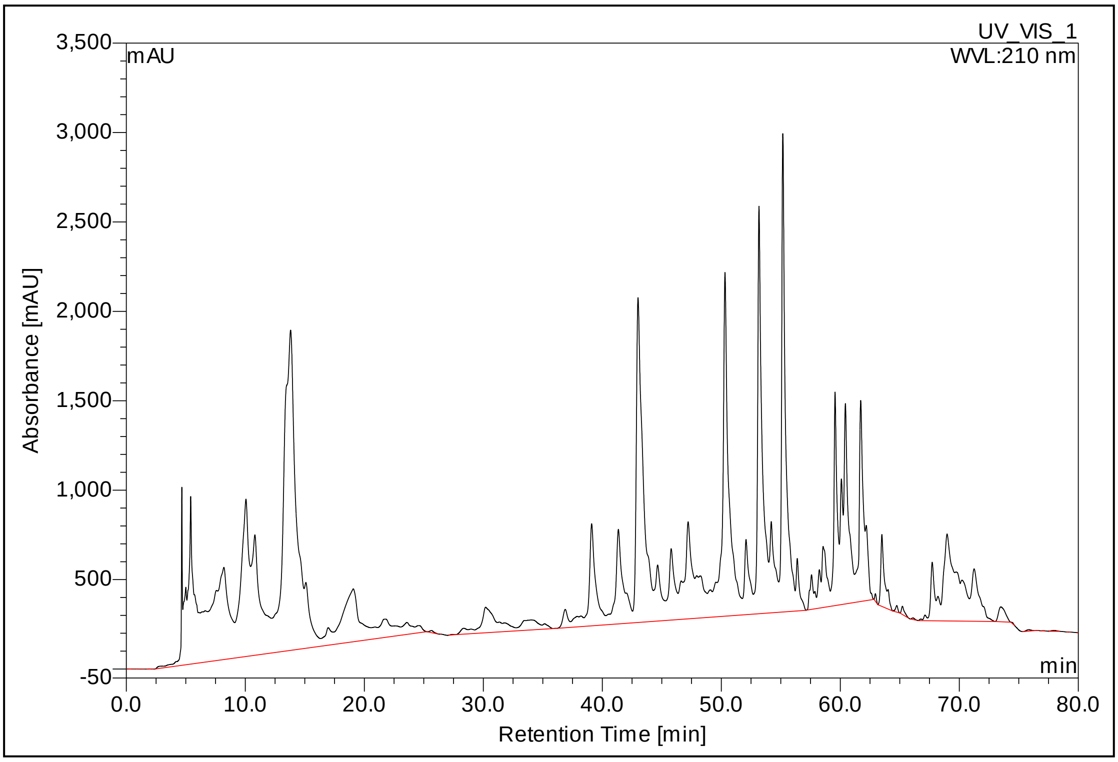

Figure S39. Purity tests of all compounds 1-18.

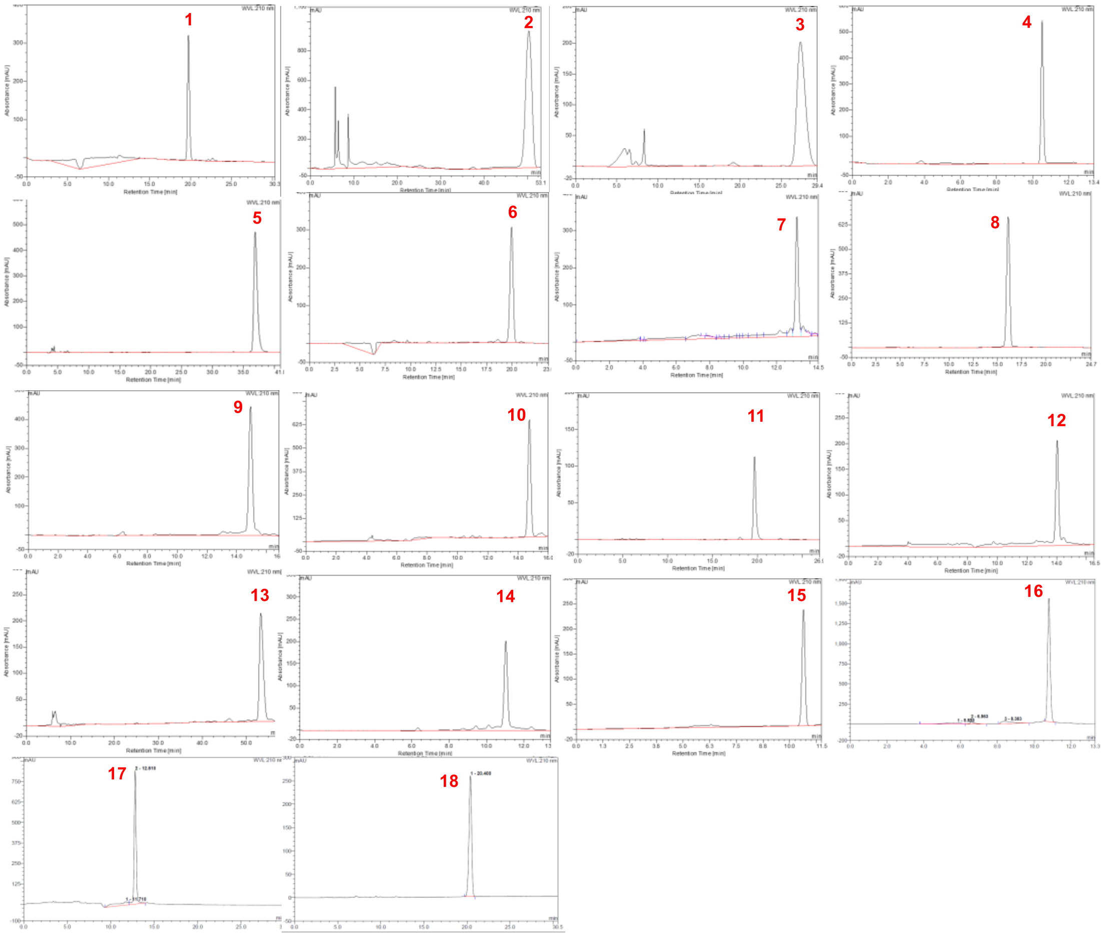

Supplement: Supplementary file 1 [file marinedrugs-19-00588-s001.zip › marinedrugs-1416094-supplementary.pdf]
